# A network view of human immune system and virus-human interaction

**DOI:** 10.3389/fimmu.2022.997851

**Published:** 2022-10-26

**Authors:** Kang Tang, Jing Tang, Jinfeng Zeng, Wei Shen, Min Zou, Chi Zhang, Qianru Sun, Xiaoyan Ye, Chunwei Li, Caijun Sun, Siyang Liu, Guozhi Jiang, Xiangjun Du

**Affiliations:** ^1^ School of Public Health (Shenzhen), Shenzhen Campus of Sun Yat-sen University, Shenzhen, China; ^2^ School of Public Health (Shenzhen), Sun Yat-sen University, Guangzhou, China; ^3^ Department of Rheumatology and Immunology, The Affiliated Drum Tower Hospital of Nanjing University Medical School, Nanjing, China; ^4^ Department of Otolaryngology, The First Affiliated Hospital of Sun Yat-sen University, Guangzhou, China; ^5^ Key Laboratory of Tropical Disease Control, Ministry of Education, Sun Yat-sen University, Guangzhou, China

**Keywords:** protein-protein interaction, gene module, immune-related gene, virus-human interaction, new gene

## Abstract

The immune system is highly networked and complex, which is continuously changing as encountering old and new pathogens. However, reductionism-based researches do not give a systematic understanding of the molecular mechanism of the immune response and viral pathogenesis. Here, we present HUMPPI-2022, a high-quality human protein-protein interaction (PPI) network, containing > 11,000 protein-coding genes with > 78,000 interactions. The network topology and functional characteristics analyses of the immune-related genes (IRGs) reveal that IRGs are mostly located in the center of the network and link genes of diverse biological processes, which may reflect the gene pleiotropy phenomenon. Moreover, the virus-human interactions reveal that pan-viral targets are mostly hubs, located in the center of the network and enriched in fundamental biological processes, but not for coronavirus. Finally, gene age effect was analyzed from the view of the host network for IRGs and virally-targeted genes (VTGs) during evolution, with IRGs gradually became hubs and integrated into host network through bridging functionally differentiated modules. Briefly, HUMPPI-2022 serves as a valuable resource for gaining a better understanding of the composition and evolution of human immune system, as well as the pathogenesis of viruses.

## Introduction

The interplays among multiple biomolecules (mRNA, proteins, metabolites, etc.) result in diverse functional and causal relationships among distinct phenotypes. The emergence and rapid advances of network biology have provided unique strategies for systematic analysis of the relationship between genotype and phenotypes, which has been widely used in the study of human diseases (1–6). The highly networked and complex characteristics of the immune system are the important molecular basis for the host to resist the invasion of pathogens, recognize and eliminate tumors and decaying apoptotic cells, and regulate the homeostasis (7–9). As an important part of the organism, the immune system does not sustain human homeostasis in isolation, but works with other systems. Hence, immune response and viral infection should be studied from the perspective of human interactome, a comprehensive map of all biological molecular interactions.

Protein-protein interactions (PPIs) play an important role in the basic biological processes of living cells, and the knowledge of the PPI network is critical for uncovering the underlying molecular mechanisms of distinct phenotypes. With the rapid development of high throughput methods, such as yeast two-hybrid assay (10) and affinity-purification mass spectrometry (AP-MS) (11), genome-wide PPI data for human have been extensively accumulated (4, 12–15). There are a few attempts to investigate the immune system from the perspective of interactome, such as the PPI network of interferon-stimulated genes (ISGs) (16, 17) and the PPI network of the immunoglobulin superfamily (IgSF) (18, 19). These studies have improved the existing knowledge of immune regulatory networks to some extent, and provided a reliable theoretical basis for clinical treatment of infectious diseases and tumors. However, a complete and reliable immune network and its extended analysis is still missing.

The selective pressure exerted by pathogens is an important driving force for the evolution of the human immune system (20–22), and the outcome of disease is determined by interactions between host factors and pathogens. Over the past few decades, a large number of virus-host protein interactions were identified by large-scale PPI experiments and included in a variety of host-pathogen interaction databases, such as HPIDB (23) and VirHostNet (24). These datasets and the corresponding systematic analyses are crucial for understanding the host response and pathogenesis due to pathogen infection. However, most efforts focused on only a few viruses, such as human immunodeficiency virus (HIV) (25), Influenza A virus (IAV) (26), human papillomavirus (HPV) (27), human cytomegalovirus (HCMV) (28, 29) and human herpesvirus (HHV) (30, 31), and emerging viruses, such as severe acute respiratory syndrome coronavirus 1 (SARS-CoV-1) (32, 33), Middle East respiratory syndrome coronavirus (MERS-CoV) (32) and severe acute respiratory syndrome coronavirus 2 (SARS-CoV-2) (33–35), which caused and are still causing the damage (36). Most previous studies focused on the analysis of specific virus-host interaction, but the multiple source of virus-human protein interactions combined with the network view could be helpful for understanding the strategies of viruses to use host cells to complete their own life cycle and at the same time evade host immune system defenses (37–40). 

In this study, HUMPPI-2022, a high-quality human PPI network, was constructed based on data integration from five primary databases (41–45), and network characteristics for the human immune system and virus-human interactions were systematically investigated. Information of the network signatures for the immune-related processes and virus-human interactions are helpful for better understanding of the molecular mechanisms of the host immune response to viral infection, and ultimately targeted prevention and curation of infectious diseases.

## Materials and methods

### Data collection

#### The human PPIs

The human PPIs were extracted from five primary source databases: BioGRID (41), DIP (42), IntAct (43), MINT (44) and MatrixDB (45). For each reported PPI, the interacting proteins were converted to gene symbol pairs using an ID mapping table downloaded on May 27, 2020 from Ensembl (http://asia.ensembl.org) (46). The following interactions were not included in this study: (1) interactions that did not have an associated PubMed ID (PMID) or a valid PSI-MI experimental interaction detection method (47, 48); (2) genetic or protein-DNA interactions; (3) PPI annotated with “invalid” evidence terms according to the previous classification method (12, 14). The starting full dataset comprises 309,733 human protein interactions validated by at least one experimental method and reported in at least one article indexed in PubMed [**Figure S1**, showed by UpSetR plot (49)].

To get the high-quality PPIs, the dataset was divided into high-throughput (HT) and low-throughput (LT) subsets based on the size of the dataset in the publication. If more than 100 PPIs were reported in that publication, it was classified as HT and otherwise as LT (50). PPIs only supported by one HT experimental method were removed. After the above filtration steps, the resulting PPI network, HUMPPI-2022, contains 78,261 interactions and covers 11,202 protein-coding genes (**Table S1**).

#### The human immune-related genes and immune-related processes

A total of 5,422 IRGs were collected from four databases, namely ImmPort (https://www.immport.org/home), InnateDB (51), Immunome (52) and PathCards (53). IR processes were collected from ImmPort, InnateDB and PathCards, and a total of 165 processes were generated after deduplication (**Table S2**).

#### The virus-human interactions

Interaction data for a variety of viral families were obtained from pathogen-host interactions databases HPIDB (23), VirHostNet (24) and from general databases BioGRID (41), DIP (42), IntAct (43) and MINT (44). Additionally, virus-human interactions for five coronaviruses, including SARS-CoV-2, were obtained from four studies (32–35). For each reported virus-human PPI, the human interacting proteins were converted to gene symbol pairs using an ID mapping table downloaded from Ensembl (46). To explore the virus-human interaction mechanism, different viral strains from same species were merged, and finally 29 viruses that target more than 200 host genes were showed (**Table S5**). Furthermore, the genes were arranged in reverse order based on the number of viruses targeting them, with the top 1% (genes were targeted by at least 14 viruses) considered as pan-viral targets.

#### Gene age data

The human gene age data was retrieved from an updated study (54). In brief, each protein-coding gene was dated and assigned to a given branch by inferring the absence and presence of orthologs along the vertebrate phylogenetic tree, based on UCSC syntenic genomic alignment. Genes without evolutionary ages were excluded from this analysis. Edge age here was assigned based on the evolutionary age of the younger gene. The divergence time of each gene age group was assigned as the middle time point for each branch and the oldest branch (branch 0) is arbitrarily set as 500 Mya (million years ago).

#### Other datasets

2,391 essential genes were taken from two previous papers (55, 56). 468 cancer driver genes were extracted from https://www.intogen.org (57).

Per-tissue median gene expression level from GTEx data portal (Release V8, dbGaP Accession phs000424.v8.p2) was downloaded on 03/02/2021. A gene was considered expressed in a given tissue if the Transcripts Per Kilobase Million (TPM) value > 1. Tissue specificity index (τ) (58) was used to measure the specificity of expression profile for a given gene.

The information of subcellular localization of 12,003 human proteins to 30 cellular structures and substructures were obtained from Thul et al. (59).

### Network topological properties analysis

Degree, clustering coefficient, assortativity and eigenvector centrality used to measure genes’ importance in HUMPPI-2022 were computed with Python NetworkX (60). Degree (or connectivity) is a basic network property, which indicates the number of neighbors of a node. Node with non-random high degree is called a hub. Clustering coefficient measures the degree of interconnectivity in the neighborhood of a node (61). Assortativity represents the average degree of the nearest neighbors of a node (62). Eigenvector centrality denotes the influence of a node in the network topology, which considers the importance of its neighbors (63).

To evaluate whether these property values of a given gene list differed from a randomly selected gene subsets of equivalent size, as Huttlin et al. (4) did, gene labels were scrambled across the network and a new average was calculated for the randomized list of genes. This process was repeated 10,000 times to define the null distributions for each statistic. Since these distributions were normally distributed, Gaussian distributions were fitted to each and used to assign *Z* scores and *P* values for each statistic associated with the true set of given genes.

### Detection and analysis of gene modules

#### Gene module detection

As described by Huttlin et al. (4), the Unsupervised Markov cluster (MCL) algorithm (64) was used to partition HUMPPI-2022 into modules of tightly interconnected genes. Based on the average functional similarity (3) of modules, the -force-connected option of y and inflation parameter of 2.28 were chosen (**Figure S2A** and **Table S3**).

#### Module-module association network

In addition to the identification of modules using MCL, interconnections between modules were also explored. First, the full set of 78,261 interactions was trimmed to include only those connecting one module with another, and the set of all module pairs connected by one or more interactions was identified. Fisher’s exact test was used to identify pairs of modules that were enriched for interactions among them, followed by multiple testing correction (65). The module-module association network including 1,936 associations at a 1% FDR (**Table S3**) was got and visualized by Cytoscape (version 3.8.0) (66).

#### IRGs enrichment of module

The hypergeometric test was used to evaluate the enrichment of IRGs, taking into account the size of the module, the size of HUMPPI-2022, and the fraction of network genes that were IRGs. Above this, 595 modules containing two or more IRGs without reaching statistically significant enrichment after a multiple testing correction (65), and only 13 modules were found to be enriched with IRGs at a 1% FDR.

To assess the tendency for modules containing IRGs or enriched for IRGs to be centrally located in the module-module association network. The Wilcoxon test was used to compare the degree distributions of modules enriched and not enriched with IRGs, and to compare the degree distributions of modules containing multiple IRGs with modules containing 0 or 1 IRG.

#### Virally-targeted modules

To understand the association between gene module and viral perturbation, targeted genes for each virus were then mapped to each module identified in HUMPPI-2022. A hypergeometric test was used to evaluate the enrichment of modules targeted by virus, taking into account the size of module, the number of virally-targeted genes (VTGs) in the module, the number of VTGs within the network and the total network size. This process was repeated for each module and for each virus. After multiple testing correction (65), those modules enriched with genes involved with each virus at a 5% FDR were identified (**Table S5**). A similar approach was used to identify specific VTMs for SARS-CoV-2, SARS-CoV-1 and MERS-CoV, where the number of specific VTGs contained in each module is considered (**Table S5**).

#### Gene ontology, IR process and subcellular localization enrichment analysis

The gene sets were tested for enrichment of gene ontology (GO) terms, IR processes and subcellular localization. The over-representation analysis was based on the hypergeometric distribution and performed using the enricher function of clusterProfiler package in R with default parameters (67). The GO terms were obtained from the c5 category of Molecular Signature Database (MSigDB v7.4) (68). Significant GO terms, IR processes and subcellular localization (adjusted *p*-value < 0.01) were identified and for multiple significant terms with Jaccard similarity ≥ 0.99, we selected the term with the lowest adjusted *p*-value. The Wilcoxon test was used to compare the degree distributions of modules enriched and not enriched with IR process.

The significant functional domains in HUMPPI-2022 were determined and visualized by the SAFE method (69) in Cytoscape (version 3.8.0) (66). GO terms for each gene were also extracted from the c5 category of MSigDB v7.4 (68). SAFE analysis was run with the default option except layout = edge-weighted spring embedded layout, neighborhoodRadius = 200, and neighborhoodRadiusType = absolute.

### Statistical analysis

R (version 4.1.2) was used for the statistical analysis, and the R package ggplot2 was used to generate most of the figures. The regression correlation and the corresponding significant test were calculated and added by R package ggpmisc.

## Results

### The landscape of human protein interactome

**Figure 1:**
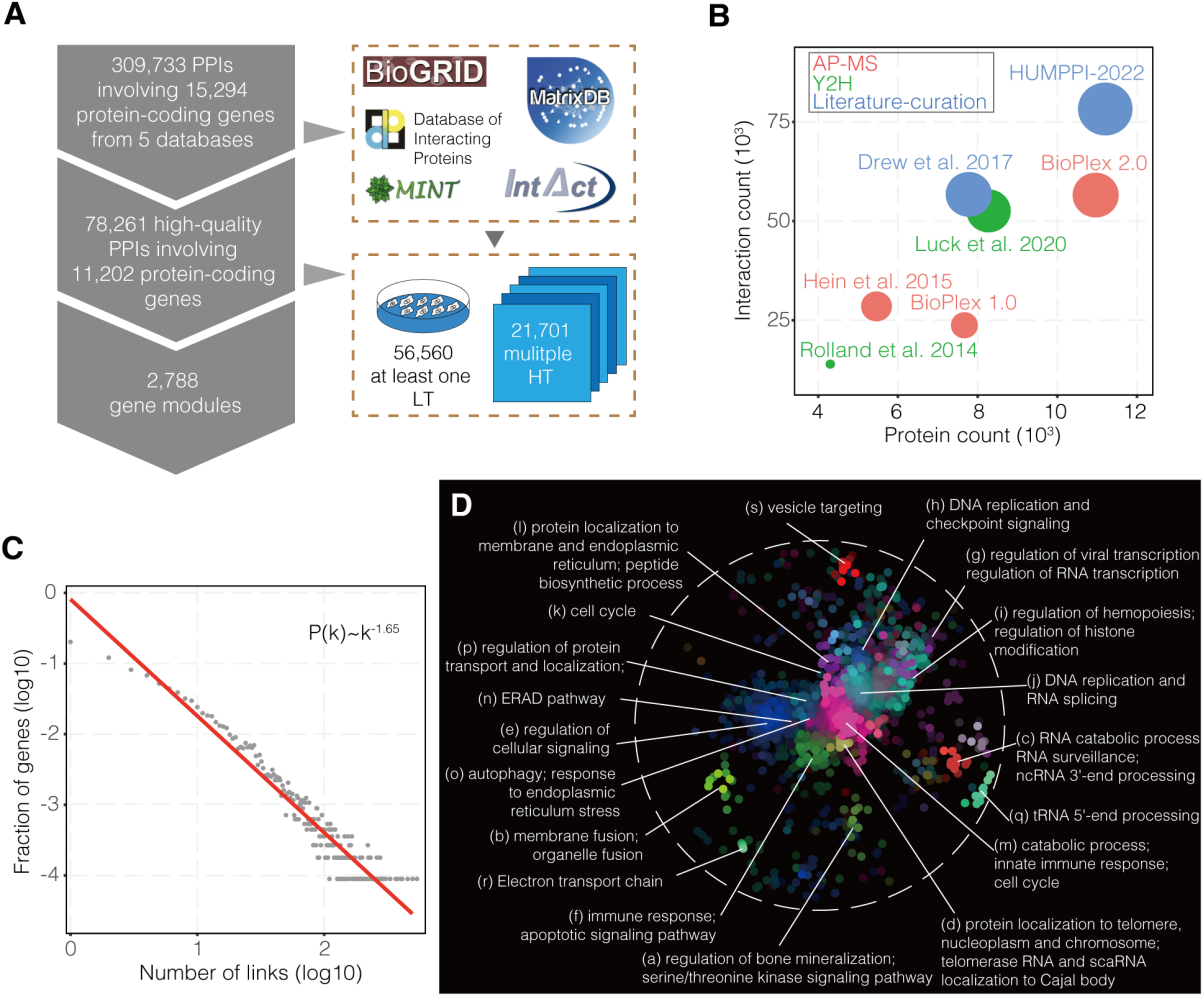
The construction of human protein-protein interaction network. **(A)** Acquisition of high-quality literature-curated PPIs and module statistics. HT, High-Throughput; LT, Low-Throughput. **(B)** Comparisons with interaction networks derived from HT approaches and literature-curation method with respect to number of protein-coding gene and interaction counts. Circle area is proportional to interaction counts, while shading denotes the experimental strategy. AP-MS, affinity-purification mass spectrometry; Y2H, yeast two-hybrid assay. **(C)** Power-law degree distribution of HUMPPI-2022. **(D)** The functional domains in HUMPPI-2022. All region-specific GO terms were combined into 19 domains based on the similarity of their enrichment landscapes. Different colors represent different functional domains.

In order to get a comprehensive while relatively high confident dataset, PPIs with high confidence, either reported by multiple high-throughput methods or supported by a single low-throughput approach, from five primary databases were integrated into HUMPPI-2022 which contains 78,261 interactions from 11,202 protein-coding genes (**Figure 1A** and **Table S1**, check Methods for more details). HUMPPI-2022 covers more genes and interactions compared to up-to-date datasets based on high-throughput approaches (4, 12–14) and literature-curation strategy (70) (**Figure 1B**). Overall, about 28.73% (21,701/78,261) of interactions were confirmed by multiple high-throughput approaches and the others each supported by a low-throughput method. HUMPPI-2022 shows an approximately scale-free topological structure (71) with a degree exponent of 1.65 (**Figure 1C**). HUMPPI-2022 covers 81.02% of the essential genes, 82.39% of the cancer driver genes, 68.68% of IRGs and 77.45% VTGs (see Methods for data source details). Functionally related genes, such as genes in the same complexes and pathways, often closely interact with each other and form tightly connected modules in the PPI network (72). MCL algorithm (64) was applied to HUMPPI-2022 and 2,788 genes modules were identified. 1,225 modules with no less than 3 genes were used for further analysis (**Figure S2B** and **Table S3**). Furthermore, 19 significant functional domains were identified in the network based on SAFE (69), and each domain was associated with a unique list of enriched GO terms (**Figure 1D**).

### The importance of IRGs in the network

**Figure 2:**
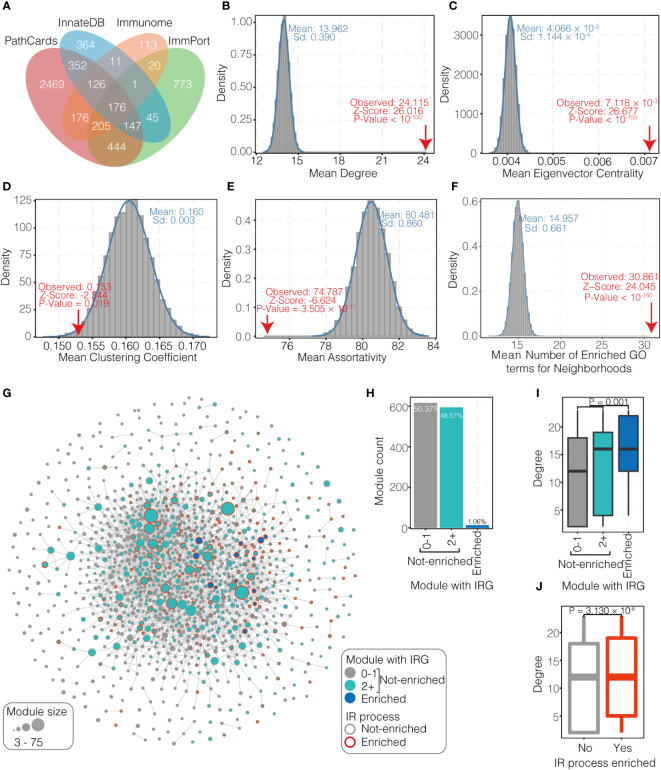
Immune-related genes (IRGs) in PPI network. **(A)** The statistics of IRGs from four databases. **(B-E)** Network properties (degree, eigenvector centrality, clustering coefficient, and assortativity, respectively) of IRGs. **(F)** Number of enriched GO terms for neighborhoods of IRGs. **(G)** Network of 1,225 modules identified through MCL clustering of HUMPPI-2022. Nodes represent distinct modules and the size reflect the gene number in each. Nodes are connected with significant link (see Methods). Green nodes mean modules containing two or more IRGs and not enriched with IRGs; Blue nodes mean modules that are enriched with IRGs (1% FDR); and modules containing less than two IRGs are colored in grey. Nodes with red border represent modules enriched to IR (immune-related) process. **(H)** Relative fractions of 1,225 modules that contain specified numbers of IRGs. **(I)** Comparison of network connectivity (degree) for modules that contain specified numbers of IRGs. **(J)** Comparison of network connectivity for modules that enriched to IR and non-IR process.

5422 IRGs from four databases as a union set were collected (**Figure 2A**) and 68.68% of them (3,724/5,422) are included in HUMPPI-2022. From the perspective of network view, IRGs are special compared to the overall proteome in several network properties (**Figures 2B–E**; *P* < 0.05). The mean degree and mean eigenvector centrality for the IRGs are significantly higher than the mean value for all the genes in HUMPPI-2022 (**Figures 2B, C**, 24.115 vs 13.962 and 7.118 × 10^-3^ vs 4.006 × 10^-3^, respectively), while the mean clustering coefficient and mean assortativity are significantly lower (**Figures 2D, E**, 0.153 vs 0.160 and 74.787 vs 80.481, respectively). A higher mean degree and mean eigenvector centrality indicate that the IRGs tend to have more interactions with other genes and may play more important roles in the network. And a lower mean clustering coefficient and mean assortativity suggest IRGs may not preferentially interact with each other. Furthermore, the mean number of enriched distinct GO terms for neighborhoods of the IRGs are significantly higher (**Figure 2F**, 30.861 vs 14.957, *P* < 10^-100^), which means IRGs tend to link various biological processes and may be pleiotropic.

Next, enrichment of IRGs in modules and preference of IRGs in the network were tested. 74.22% of IRGs in the network (2,764/3,724) reside within one of the 905 modules with no less than 3 genes (totally 1225 modules with no less than 3 genes, **Figure S2B** **).** Among these modules, only 1.06% (13) are enriched with IRGs (**Figures 2G, H** **)**. 595 modules (48.57% among 1225) contained two or more IRGs without reaching statistically significant enrichment (**Figures 2G, H** **)**. Functional enrichment analysis reveals that modules enriched for IRGs are all involve in IR processes (**Table S4**), such as pattern recognition receptor signaling pathway (module #31), response to chemokine (module #36), antigen processing and presentation (module #88), cytokine mediated signaling pathway (module #209), and complement pathway (module #270). In addition to the 13 IRGs-enriched modules, another 199 modules were also found to be enriched with IR processes (**Table S4**), and all those modules enriched with IR processes show higher connectivity in the module-module association network (**Figures 2G, I, J**).

### Network view of virus-human interaction

Understanding the physical interaction between viral and host genes from the perspective of network view could facilitate our understanding for the pathogenesis of viruses. In order to get into this problem, 23,832 unique interactions that involve 7,649 human genes and 226 viruses from six databases were collected. In this study, 29 viruses which target more than 200 host genes were showed below (**Figure S3B** and **Table S5**).

**Figure 3:**
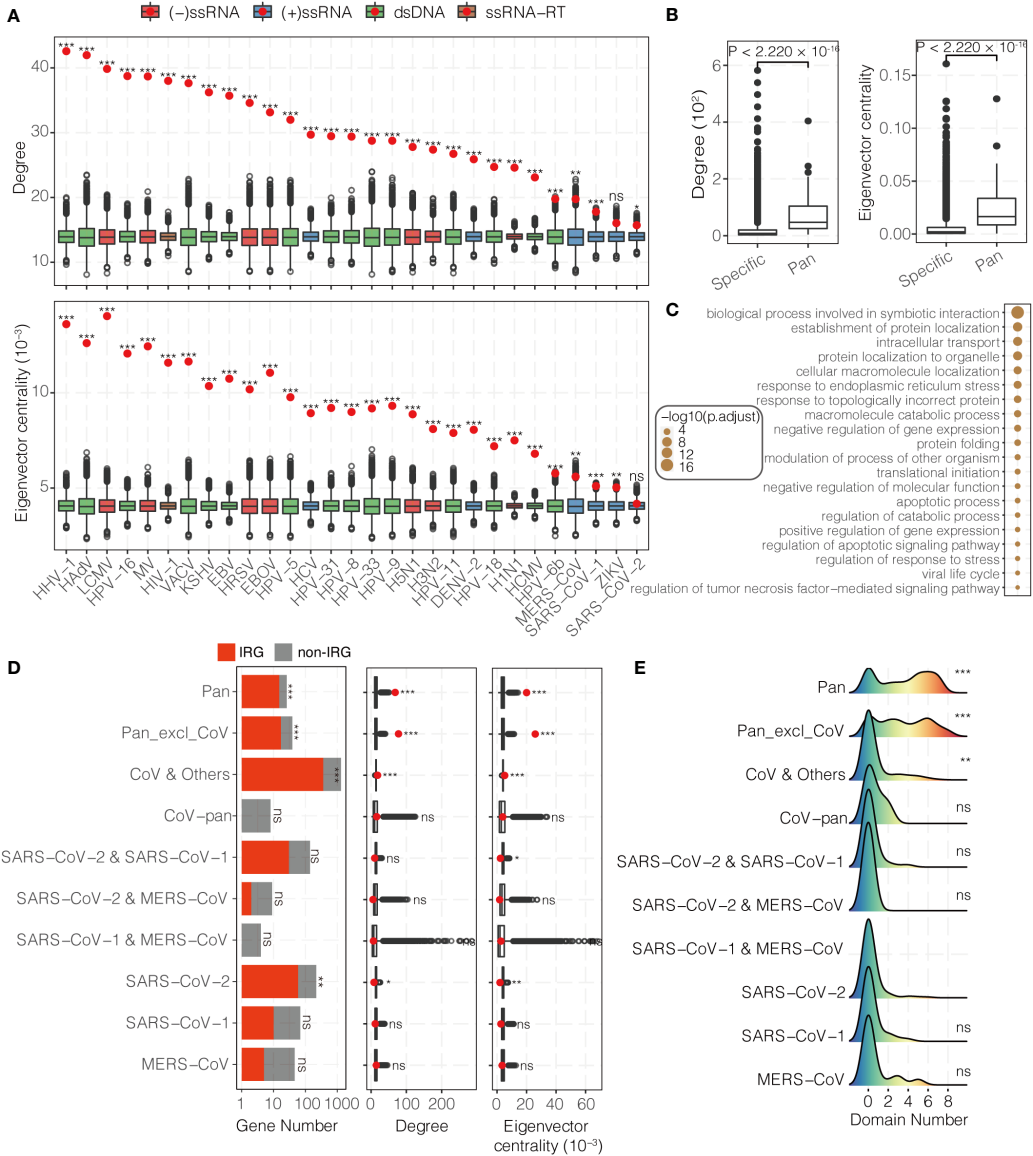
Topological and functional characteristics of virally-targeted human genes. **(A)** Degree (upper) and eigenvector centrality (bottom) of VTGs. The red dots and the corresponding boxes represent the observed values and the simulated distributions using the bootstrapping method, respectively. DENV-2, Dengue virus 2; EBOV, Ebola virus; EBV, Epstein-Barr virus; HAdV, Human adenovirus C; HCMV, Human cytomegalovirus; HCV, Hepatitis C virus; HHV-1, Human herpesvirus 1; HIV-1, Human immunodeficiency virus 1; HPV-5, HPV-6b, HPV-8, HPV-9, HPV-11, HPV-16, HPV-18, HPV-31 and HPV-33, Human papillomavirus 5, 6b, 8, 9, 11, 16, 18, 31 and 33; HRSV, Human respiratory syncytial virus; H1N1, H3N2 and H5N1, Influenza A H1N1, H3N2 and H5N1 virus; KSHV, Kaposi’s sarcoma-associated herpesvirus; LCMV, Lymphocytic choriomeningitis virus; MV, Measles virus; MERS-CoV, Middle East respiratory syndrome coronavirus; SARS-CoV-1, severe acute respiratory syndrome coronavirus 1; SARS-CoV-2, severe acute respiratory syndrome coronavirus 2; VACV, Vaccinia virus; ZIKV, Zika virus. **(B)** Degree (left) and eigenvector centrality (right) distributions of specific- and pan-viral targets. **(C)** Functional enrichment analysis of pan-viral targets. **(D)** Number (left), degree (middle) and eigenvector centrality (right) of three coronaviruses targeted host genes. The red dots and the corresponding boxes represent the observed values and the simulated distributions using the bootstrapping method, respectively. Pan, pan-viral genes targeted by at least one coronavirus; Pan_excl_CoV, pan-viral genes not targeted by coronavirus; CoV & Others, host genes targeted by at least one coronavirus and at least one other virus; CoV-pan, host genes only targeted by all three coronaviruses; SARS-CoV-2 & SARS-CoV-1, SARS-CoV-2 & MERS-CoV and SARS-CoV-1 & MERS-CoV, three coronaviral pairs of targeted host genes; SARS-CoV-2, SARS-CoV-1 and MERS-CoV, specifically targeted host genes for each coronavirus. **(E)** Functional domain number of three coronaviruses targeted host genes. ***P-value < 0.001, **P-value < 0.01, *P-value < 0.05, ns, not significantly different.

An efficient way for viruses to hijack host is targeting the hubs of host network (37, 38). In order to test this, VTGs were mapped to the HUMPPI-2022. In comparison with general human genes, the VTGs have higher degree and centrality (**Figure 3A**). 65 genes (see Methods) that were targeted by at least 14 viruses were identified, and further analysis indicates that those pan-targeted genes are with even higher degree and centrality (**Figure 3B**; Wilcoxon rank sum test, *P* < 2.220 × 10^-16^), and enriched in fundamental functions (**Figure 3C** and **Table S6**) and essential genes (**Figure S3C**; hypergeometric test, *P* = 1.914 × 10^-22^).

For a special case, functions and network topological properties for the host genes targeted by coronavirus were explored. Compared with genes targeted by multiple viruses, the 131 SARS-CoV-2 specific targets (totally 1,036 SARS-CoV-2 targets in the network) are different in following ways: they are not hubs and not in a central position in the network (**Figure 3D**); they are only enriched in one biological process, namely nucleotide phosphorylation (**Figure S4** and **Table S6**); they are not enriched in any IR processes even they were significantly enriched in IRGs (**Figure 3D** and **Table S6**); they are not enriched in any cellular structures (**Table S6**); only 7.63% (10/131) reside within one of the 5 SARS-CoV-2 VTMs (totally 23 SARS-CoV-2 VTMs, **Figure S5A** and **Table S5**), and only module #43 is enriched with SARS-CoV-2 specific targets (**Figure S5B** and **Table S5**; hypergeometric test, *P* = 8.825 × 10^-6^); they are less likely to locate in functional domains than pan-targeted genes (including Pan and Pan_excl_CoV) and genes targeted by different families of viruses (**Figure 3E** and **Table S5**; Wilcoxon rank sum test, *P* < 0.01). These phenomena can be also found in SARS-CoV-1 and MERS-CoV (**Figures 3D, E** **)**.

### Evolutionary perspective of IRG and VTG

**Figure 4:**
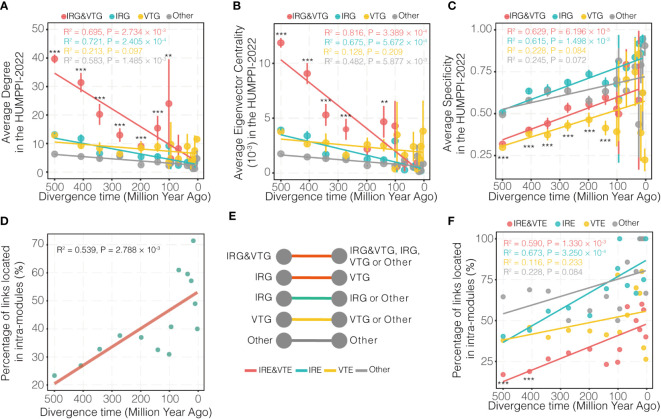
Evolutionary pattern of genes and edges related to their divergence times. **(A–C)** Distribution of PPI network degree, eigenvector centrality and tissue expression specificity for four categories of genes from different phylogenetic branches. Virally-targeted immune-related genes (IRG&VTGs), immune-related genes (IRGs), Virally-targeted genes (VTGs) and other genes (Others) are highlighted in red, cyan, yellow and grey, respectively. **(D)** Evolutionary pattern of edges related to their divergence times. **(E)** Schematic diagram of four categories of edges. Virally-targeted immune-related edges (IRE&VTEs), immune-related edges (IREs), virally-targeted edges (VTEs) and other edges (Others) are highlighted in red, cyan, yellow and grey, respectively. **(F)** Evolutionary pattern of four categories of edges related to their divergence times. IRE&VTEs, IREs, VTEs and Others are highlighted in red, cyan, yellow and grey, respectively. The divergence time of each gene age group is assigned as the middle time point for each branch. The oldest branch (branch 0) is arbitrarily set as 500 Mya. *** *P*-value < 0.001, ** *P*-value < 0.01.

Along the evolution, new genes were generated and were integrated into the network to gradually take their function roles. In order to look into the age effect of genes, role of new genes was explored from the view of network and under the consideration of their relationship with IRGs and VTGs. First of all, ages for each node and edge were labelled in HUMPPI-2022. These age labels were determined by genes that originated in every period of evolution along the well-resolved phylogeny of vertebrates (**Figure S6**), retrieved from an updated dataset (54). Genes were categorized into virally targeted IRGs (IRG&VTGs), immune-related genes but not VTGs (IRGs), Virally-targeted genes but not IRGs (VTGs) and other genes (Others), and the connectivity and centrality of them were calculated, which shows that IRG&VTGs have more links and are more centrally located in the network than non-IRG&VTGs (**Figure S7**; Wilcoxon rank sum test, *P* < 0.001), and in a time-dependent manner (**Figures 4A, B**; > 140 Mya, Wilcoxon rank sum test, *P* < 0.01). Additionally, compared to non-immune-related genes, we found that immune-related genes (including IRGs and IRG&VTGs) gradually evolved broader expression patterns (**Figure 4C**; > 140 Mya, Wilcoxon rank sum test, *P* < 0.001).

Given the observation that new genes experienced a gradual integration process into PPI network, their roles from the perspective of links (or interactions) and view of functional modules was also explored. To do so, the percentage of interactions with different phylogenetic age within modules in HUMPPI-2022 were investigated. Results shows that genes gradually established links between modules (**Figure 4D**), indicating that new PPIs contributed to the formation of modules, and more interactions appeared between modules over time and possibly related to the collaboration of biological processes. Furthermore, to explore whether virus targets an intra-module or inter-module edge, edges were classified as virally-targeted immune-related edges (IRE&VTEs), immune-related edges (IREs), virally-targeted edges (VTEs) and other edges (Others) (**Figure 4E**) and compared to each other. Percentage distributions of intra-module edges for each category of edges show that IRE&VTEs originated 400 Mya tend to connect modules more (**Figure 4F**; hypergeometric test, *P* < 0.001).

## Discussion

The immune system is highly efficient and relies on the many genes working together to defend pathogens. Theoretical advances in network science and paralleling advances in high-throughput methods have provided a framework to interpret complex phenotypes of human. A high-quality human PPI network makes it possible to learn the immune responses and virus-host interactions from the angle of systems biology. Here, we constructed HUMPPI-2022, a systematic human protein interactome map with more than 70,000 PPIs of high biophysical quality (**Figure 1** and **Table S1**). In contrast to previous studies that focused on limited viruses (37, 73) or isolated states of host factors (74), the network we constructed, combined with IRGs and VTGs, not only makes it possible to understand the molecular mechanism of the immune response and viral pathogenesis, but also provides a unique angle to study the evolutionary pattern of the immune system.

First of all, network topologies for IRGs were analyzed, with most IRGs as hubs and located in the center of the network (**Figures 2B, C** **)**. Interestingly, the lower graph assortativity and a greater number of neighborhoods enriched GO terms of the IRGs (**Figures 2E, F** **)** indicate that the IRGs would interact with other types of genes. According to the statistics of the number of IRGs contained in modules, it was found that only 1.06% of modules enriched with IRGs (**Figure 2H**), which further proved that IRGs were involved in diverse biological processes. Since the network centrality (75) and tissue expression patterns at mRNA expression level (76) can reflect the acquirement of pleiotropic functions, here we found immune-related genes, including IRGs and IRG&VTGs, gradually evolved into a broader expression patterns (**Figure 4C**), indicating that immune-related genes gradually acquired pleiotropic functions.

The pan-viral targets are enriched in functions known to be fundamental in biological processes during viral infection, such as protein localization, apoptotic process and regulation of gene expression (**Figure 3C**). However, these pan-viral targets are hardly enriched in immune-related functions. And specific targets of SARS-CoV-2 were not enriched in any IR processes even they were significantly enriched in IRGs and not enriched in any cellular structures (**Figure 3D** and **Table S6**). This finding suggests that viruses target immune components in diverse ways, possibly is a result of different adaptive strategies to the selective pressures from the host. Pavel et al. (77) assumed that the physically interacting proteins are host first responders to SARS-CoV-2 infection, and differentially expressed genes enriched in immune system related pathways are downstream effectors of host response. In this study, we also explored the influence of pathogens on host pathways at the module level, and found seven VTMs are involved in IR processes (**Figure S5A**), such as SARS-CoV-2 non-structural protein 16 (NSP16) targeting module #43 (**Figure S5B**), which is the only module enriched with SARS-CoV-2 specific targets and related to NF-κB signaling pathway. With the development of high-throughput technology and computational tool, a complete human interactome will provide us with a powerful way to interpret the infectious immune network in a perspective of gene modules.

The emergence of new genes is one of the most important factors in genomic evolution and genetic differences between species (78). The origin of new genes is a highly dynamic process. A previous study found that new genes “born” at a specific evolutionary node and continuously integrated into the original gene network at a rapid rate and gradually occupied a central position in the network, suggesting they may be related to speciation or adaptation evolution (76). Here, we found that IRG&VTGs occupied the core position of the network with the fastest rate in evolution (**Figures 4A, B** **)**, indicating that the immune system may evolve and become essential in the arms race with pathogens. On the other hand, new genes tend to form functional modules with similar functional genes in the early stage (**Figure 4D**). As time goes by, genes will gradually interact with other modules, and eventually form a module associated network of cooperation among different functions (**Figure 4D**). Viruses tend to influence links between modules (**Figure 4F**), so we hypothesize that viruses achieve immune evasion by disrupting the cooperation between functionally important modules.

Although HUMPPI-2022 help to expand our knowledge of human immune system and viral targets, our understanding of the interactome remains incomplete. First of all, more high-quality interaction data are needed to improve the comprehensive map. Secondly, the interactome map is a highly dynamic process, with nodes and their links changing in different tissues (79), cell lines (15) and periods of viral infection, which greatly increase the difficulty of the construction and analysis of the interaction network. The identification of spatiotemporal dynamics of interactions not only expands the interactome, but also helps us to better understand the relationship between genotype and phenotype. Thirdly, only the viral targeting on PPI network was analyzed, while the complex phenotypes reflect changes at different network layers (gene co-expression network, PPI network, metabolic network, transcriptional regulatory network, etc.). So, the combination of multiple context-specific network will be useful to fully understand the immune response and virus-host interaction, and helpful for developing precise prevention and treatment strategies in the future.

## Data availability statement

The original contributions presented in the study are included in the article/**Supplementary Material**. Further inquiries can be directed to the corresponding author.

## Author contributions

XD and KT designed the study. KT collected the data and performed the analysis. KT, JT, JZ, WS, CL, CS, SL, GJ and XD interpreted the data. KT and JZ prepared the manuscript. KT, JT, JZ, WS, MZ, QS, XY, CL, CS, SL, GJ and XD edited the paper. All authors contributed to the article and approved the submitted version.

## Funding

This work was supported by the Shenzhen Science and Technology Program under Grant KQTD20180411143323605, JSGG20200225152008136 and GXWD20201231165807008, and Guangdong Frontier and Key Tech Innovation Program under Grants 2019B020228001, 2019B111103001, 2021A111112007 and 2022B1111020006.

## Acknowledgments

We gratefully acknowledge all the authors from the original laboratories who submitted and shared data, on which this study is based.

## Conflict of interest

The authors declare that the research was conducted in the absence of any commercial or financial relationships that could be construed as a potential conflict of interest.

## Publisher’s note

All claims expressed in this article are solely those of the authors and do not necessarily represent those of their affiliated organizations, or those of the publisher, the editors and the reviewers. Any product that may be evaluated in this article, or claim that may be made by its manufacturer, is not guaranteed or endorsed by the publisher.
