# Supplementary material for: A network view of human immune system and virus-human interaction
Source: Front Immunol. 2022 Oct 26;13:997851. doi: 10.3389/fimmu.2022.997851 (PMC9643829; doi:10.3389/fimmu.2022.997851)
Supplement: Supplementary file 1 [file DataSheet_1.pdf]

Supplementary materials

Table S1. High-quality PPIs acquired from five public databases

Table S2. Modules identified by MCL algorithm and module association network

Table S3. GO terms and IR processes enriched in each module

Table S4. Virally-targeted genes (VTGs) and modules (VTMs)

Table S5. GO terms enriched in each VTG set

Table S6. The collected immune-related (IR) processes

Figure S1. The data source of literature-curated PPIs

Figure S2. Module detection via MCL clustering

Figure S3. Landscape of virus-human interactions

Figure S4. Functional enrichment analysis of virus-specific targeted host genes

Figure S5. VTMs of three coronaviruses

Figure S6. Origination of human genes

Figure S7. Topological and expression characteristics of four categories of genes

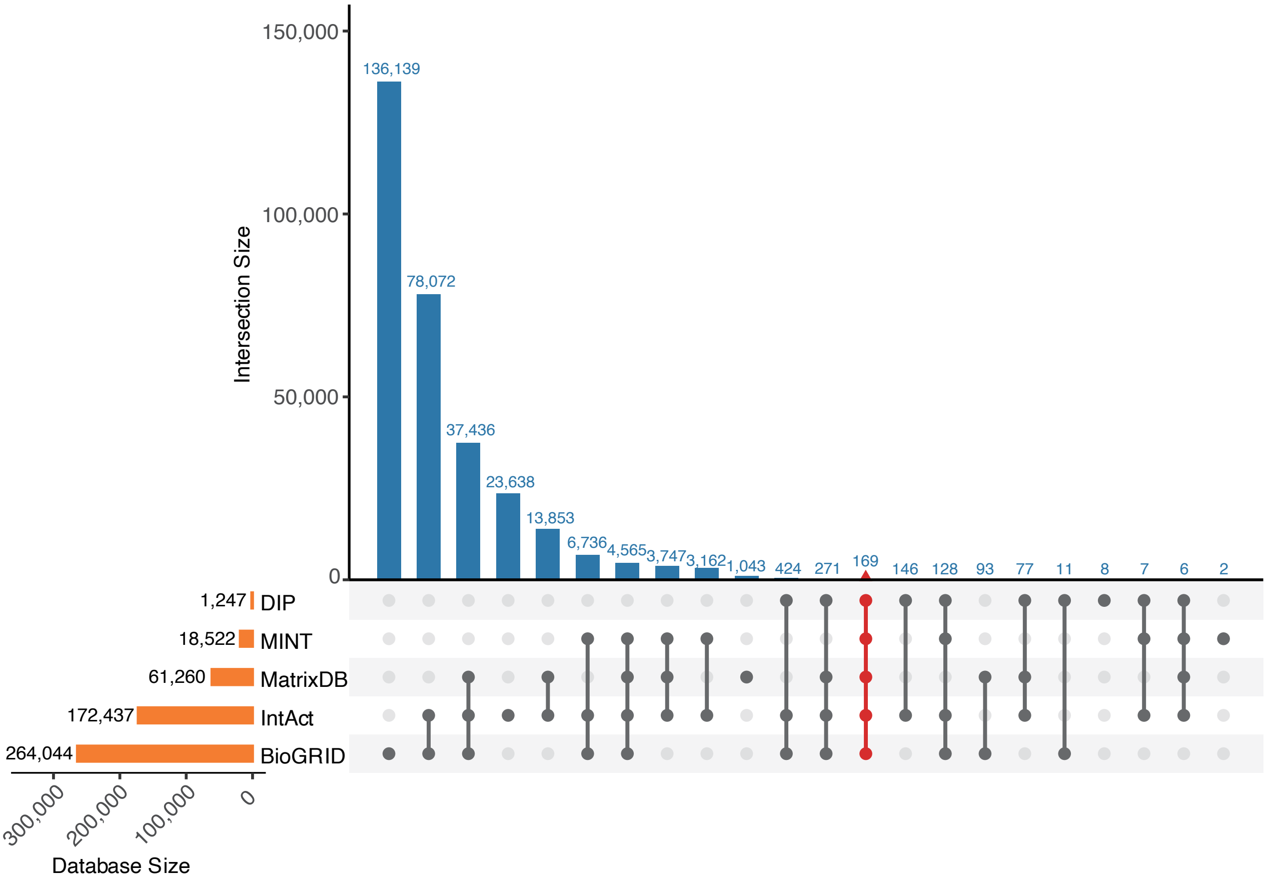

Figure S1. The data source of literature-curated PPIs. An UpSetR plot of PPIs across five primary source databases.

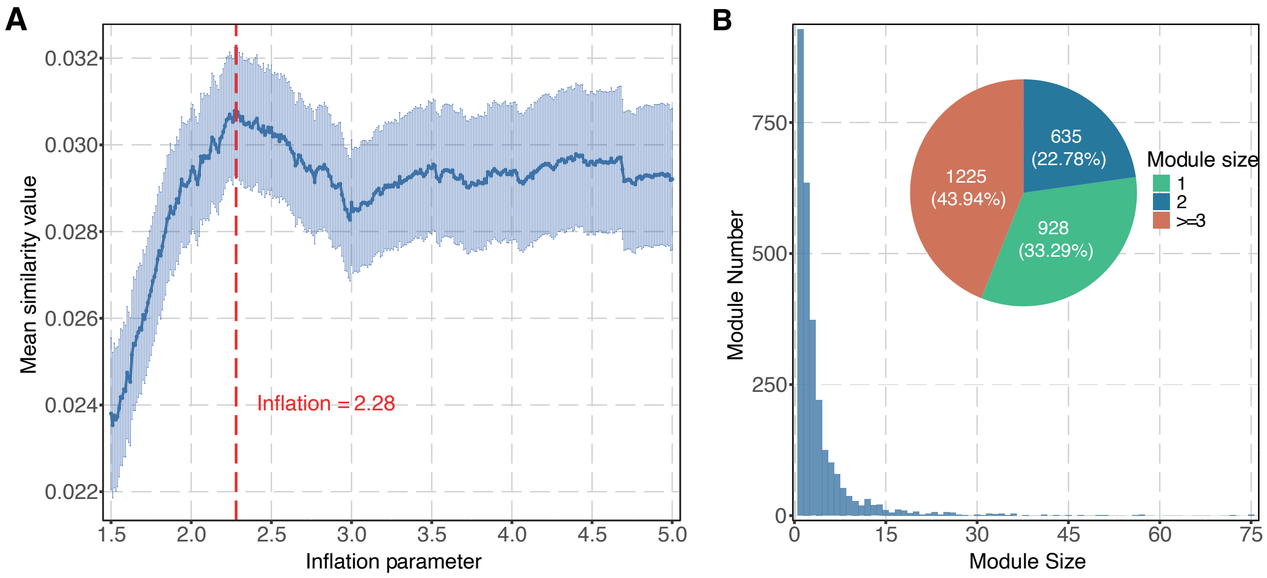

Figure S2. Module detection via MCL clustering. (A) The mean functional similarity of modules under different inflation parameter, and 2.28 was selected for the optimal parameter. (B) The module size distribution.

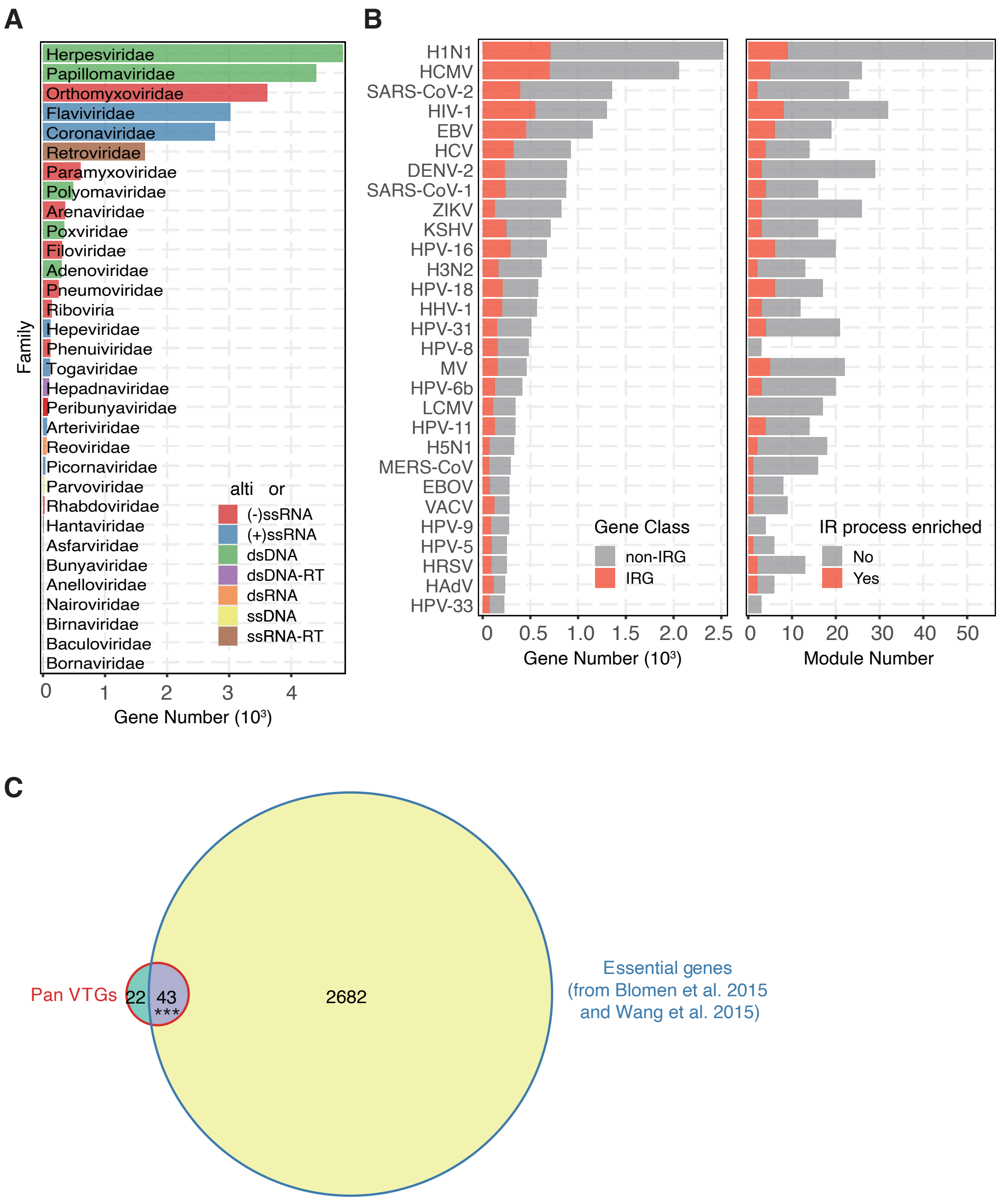

Figure S3. Landscape of virus-human interactions. (A) Number of targeted genes for each family. Colored bars correspond to different Baltimore classification. (B) Number of VTGs (left) and statistically significant VTMs (right) related to twenty-nine selected viruses. (C) Overlap between pan-viral targets and essential genes.

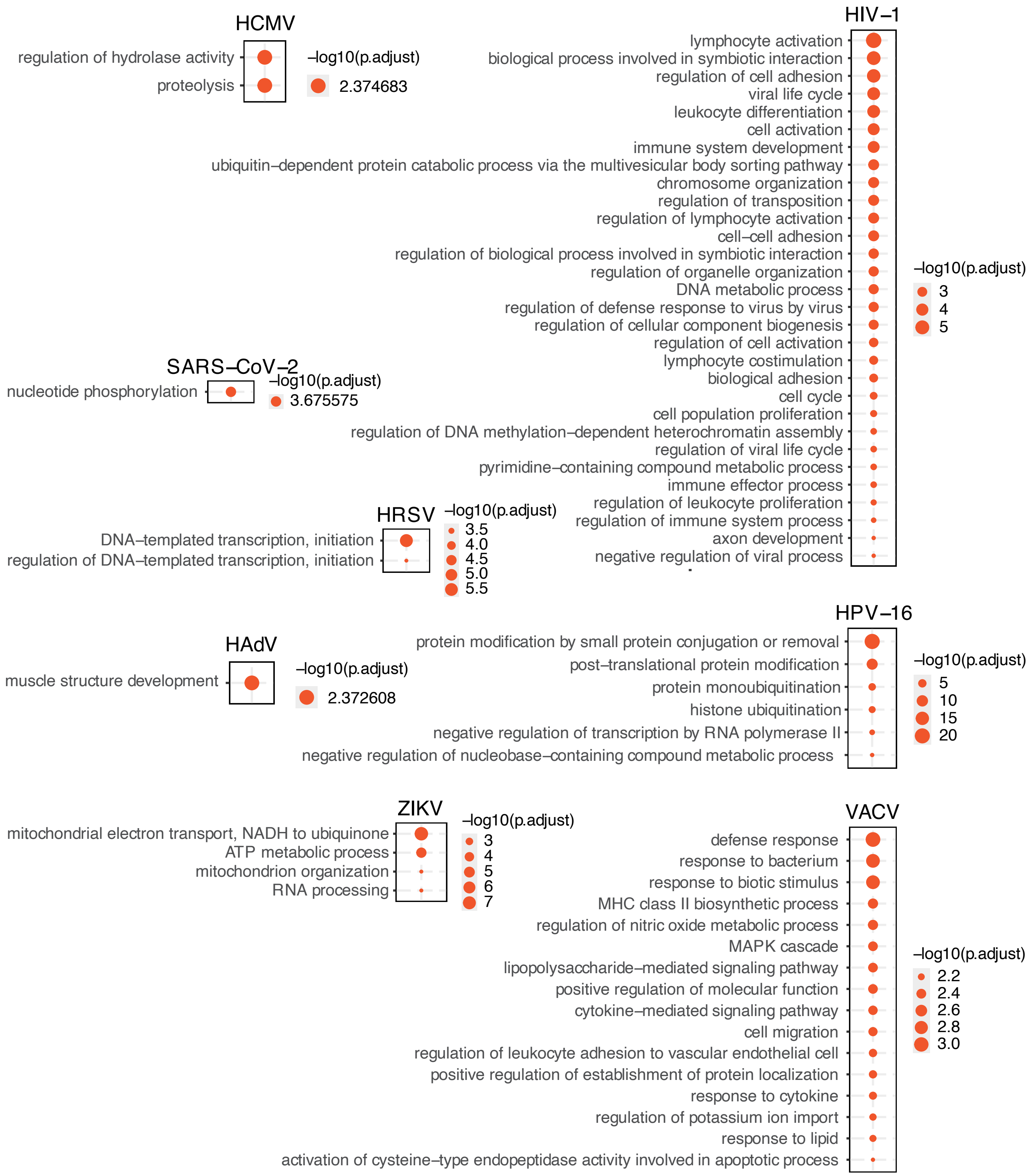

Figure S4. Functional enrichment analysis of virus-specific targeted host genes.

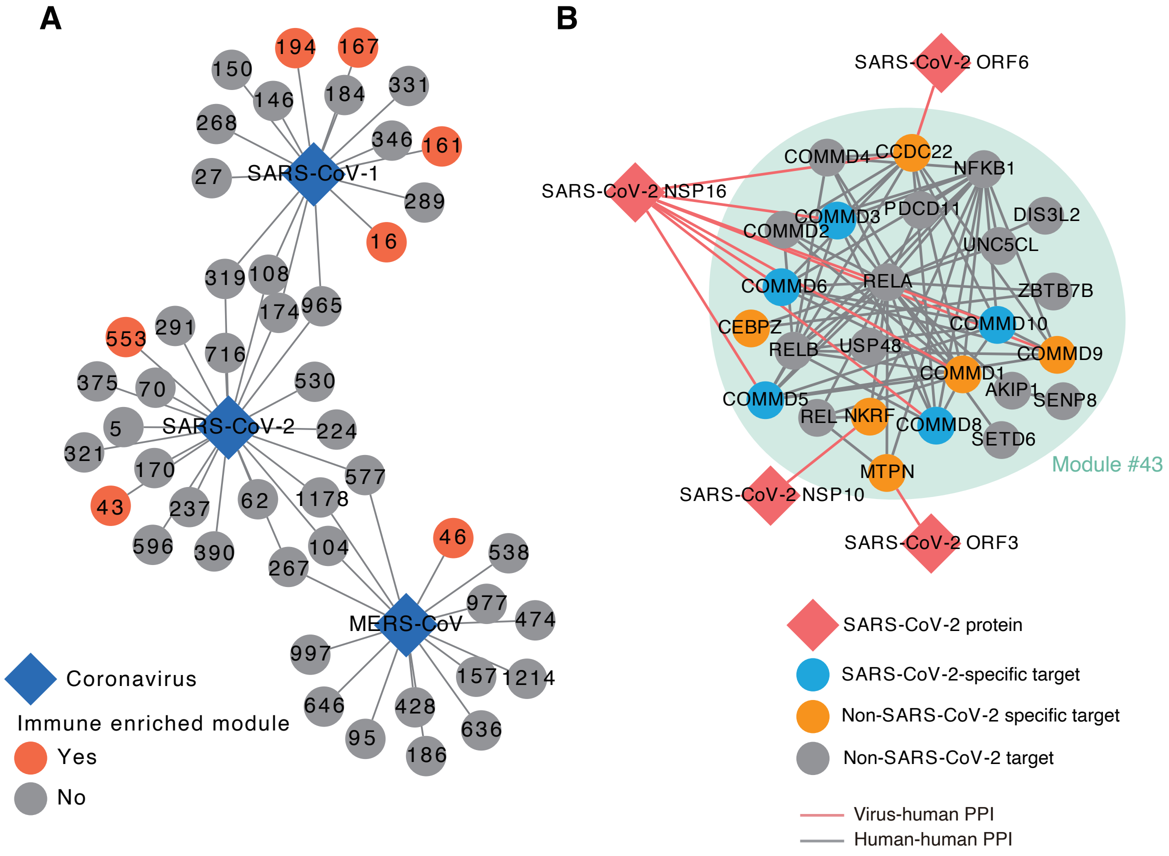

Figure S5. VTMs of three coronaviruses. (A) Associations between 3 coronaviruses (blue diamonds) and 47 gene modules (orange circles: immune enriched modules; grey circles: non-immune enriched modules). (B) Interactions between 4 SARS-CoV-2 proteins (red diamonds) and 11 human proteins (blue circles: SARS-CoV-2-specific targets; yellow circles: non-SARS-CoV-2-specific targets; grey circles: non-SARS-CoV-2 targets) in module #43. Virus-human and human-human PPIs are highlighted in red and grey lines, respectively.

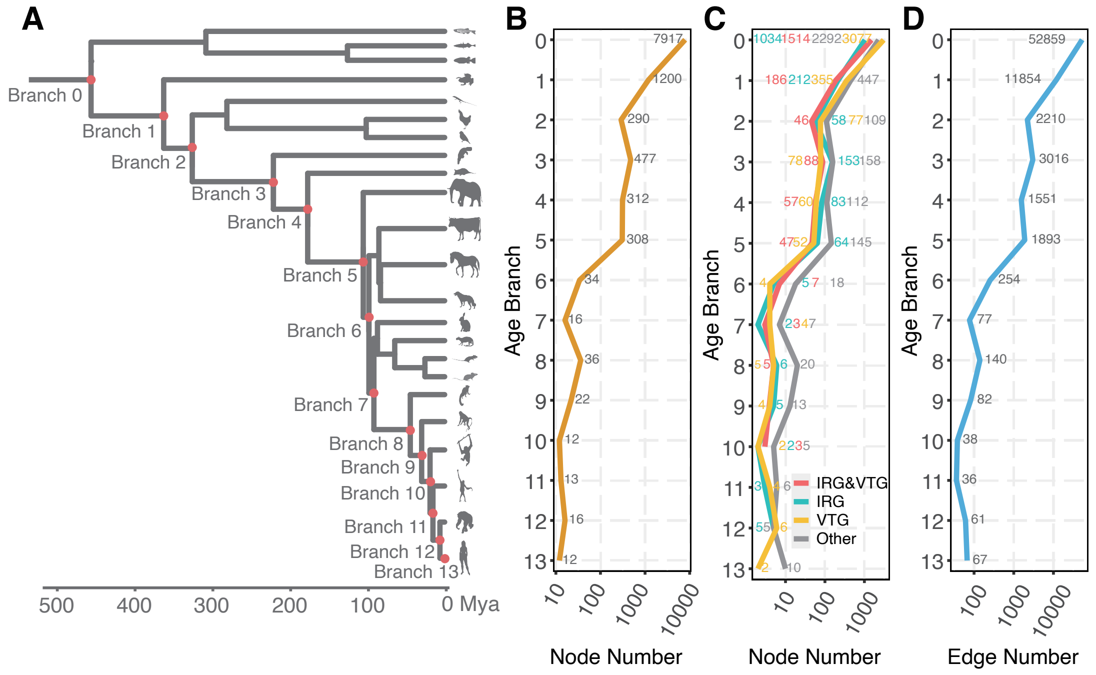

Figure S6. Origination of human genes. (A) Phylogenetic tree of vertebrates towards human together with branches and divergence times. (B) The number of genes within HUMPPI-2022 originating at each phylogenetic branch. (C) The number of four categories of genes within HUMPPI-2022 originating at each phylogenetic branch. (D) The number of edges originating at each phylogenetic branch.

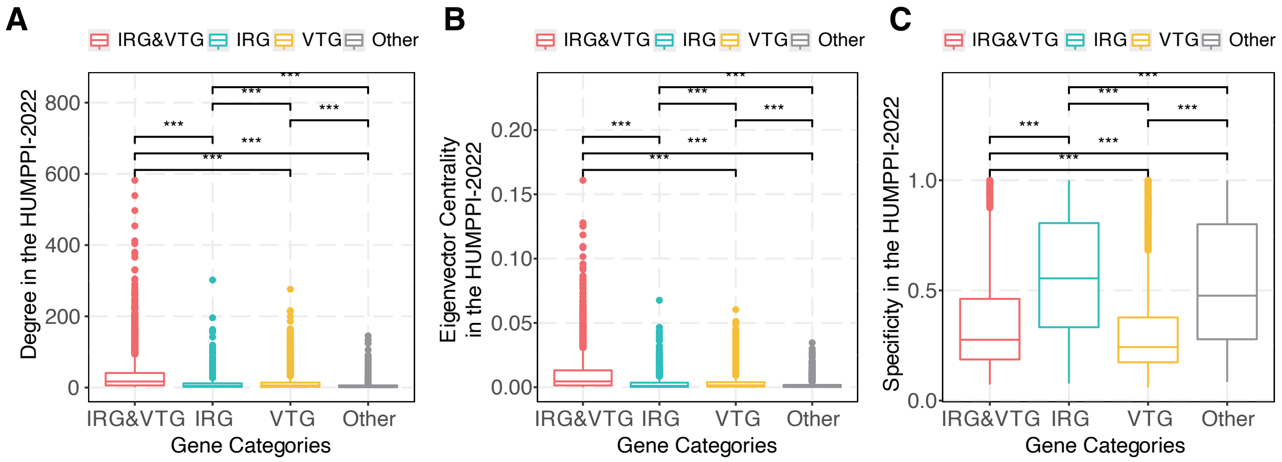

Figure S7. Topological and expression characteristics of four categories of genes. (A) - (C) Distribution of PPI network degree (A), eigenvector centrality (B) and tissue expression specificity (C) for four categories of genes. Virally-targeted immune-related genes (IRG&VTGs), immune-related genes (IRGs), Virally-targeted genes (VTGs) and other genes (Others) are highlighted in red, cyan, yellow and grey, respectively. ***P < 0.001.
